# Coumarin- and Carboxyl-Functionalized Supramolecular Polybenzoxazines Form Miscible Blends with Polyvinylpyrrolidone

**DOI:** 10.3390/polym9040146

**Published:** 2017-04-21

**Authors:** Ruey-Chorng Lin, Mohamed Gamal Mohamed, Tao Chen, Shiao-Wei Kuo

**Affiliations:** 1 Department of Materials and Optoelectronic Science, National Sun Yat-Sen University, Kaohsiung 80424, Taiwan; rueyclin01@gmail.com (R.-C.L.); mgamal.eldin12@yahoo.com (M.G.M.); 2 Key Laboratory of Graphene Technologies and Applications of Zhejiang Province, Ningbo Institute of Material Technology and Engineering, Chinese Academy of Science, Zhongguan West Road 1219, Ningbo 315201, China; tao.chen@nimte.ac.cn

**Keywords:** polymer blend, hydrogen bonding, thermal curing, polybenzoxazine, photo-responsive

## Abstract

In this study, we synthesized a novel multifunctional benzoxazine monomer (Coumarin-COOH BZ), possessing both coumarin and COOH groups, through the reaction of 4-methyl-7-hydroxycoumarin, 4-aminobenzoic acid, and paraformaldehyde in 1,4-dioxane, with the structure confirmed using ^1^H and ^13^C nuclear magnetic resonance and Fourier transform infrared (FTIR) spectroscopy. Differential scanning calorimetry (DSC), FTIR spectroscopy, and thermogravimetric analysis were then employed to monitor the thermal curing behavior of Coumarin-COOH BZ and its blends with poly(*N*-vinyl-2-pyrrolidone) (PVP), both before and after photodimerization of the coumarin moieties. DSC revealed a single glass transition temperature for each Coumarin-COOH BZ/PVP blend composition; a large positive deviation based on the Kwei equation suggested that strong hydrogen bonding existed between the Coumarin-COOH BZ and PVP segments, confirmed through FTIR spectroscopic analyses. The thermal properties improved (i.e., increased glass transition and thermal degradation temperatures) as a result of the increased crosslinking density after photodimerization under UV exposure.

## 1. Introduction

Polybenzoxazines (PBZs) are interesting materials for academic research and industrial applications because of their high thermal stability, low flammability, low surface free energy, high chemical resistance, flexible molecular structural design, and low water absorption [1,2,3,4]. The ring opening polymerization of benzoxazine (BZ) monomers occurs through thermal heating, without the need for an added catalyst and without the release of byproducts [5]. Nevertheless, PBZs do have some unwanted properties, including high process and high thermal curing temperatures [1,2,3].

The physical characteristic of PBZs can be improved using three main approaches. First, through the addition of inorganic materials (e.g., clay [6], polyhedral oligomeric silsesquioxane (POSS) [7,8], carbon nanotubes [9,10,11,12], graphene [13]) to form PBZ nanocomposites. Second, through use of functionalized BZ monomers presenting, for example, allyl, vinyl, pyrene, nitrile, methacryloyl, benzoxazole, and carboxylic acid groups [14,15,16,17,18,19,20]. Third, through blending with other thermal plastics or thermoset resins (e.g., poly(*N*-vinyl-2-pyrrolidone) (PVP), polyurethane, epoxy resin) [21,22,23]. As an example of the second approach, the Azo-COOH BZ monomer contains both azobenzene and COOH units, with the latter serving as a catalyst that lowers the curing temperature relative to that of traditional BZ monomers [11].

Coumarin is a light-responsive molecule that undergoes photo-induced dimerization through [2π + 2π] cycloaddition to form cyclobutane rings and, thereby, crosslinked structures [24]. The behavior of coumarin chromophores can change in response to variations in its chemical structure or the light source [25,26]. Two significant absorption bands appear in the UV–Vis spectra of coumarins: at 250–300 nm, representing a π-to-π* transition of the electrons of the conjugated π-system, and at 310−340 nm, representing an n-to-π*-like transition of the electrons of the C=O group [27]. Yagci et al. were the first to report that incorporating coumarin units into BZ monomers enhanced the thermal stability of the thermally cured BZ after photodimerization of its coumarin moieties [28]. We have also reported a bifunctional BZ monomer containing coumarin and pyrene moieties; its glass transition temperatures (*T*_g_) also increased after photodimerization of the coumarin moieties [12]. Ishida et al. also proposed the BZ monomer containing both coumarin unit and investigated the possible chemical structure of this new type of BZ monomer [29]. In an unrelated study, we found that miscibility could also result in large deviations in the values of *T*_g_ when strong hydrogen bonding existed between phenolic OH groups of PBZ and the C=O groups of PVP [21].

**Scheme 1:**
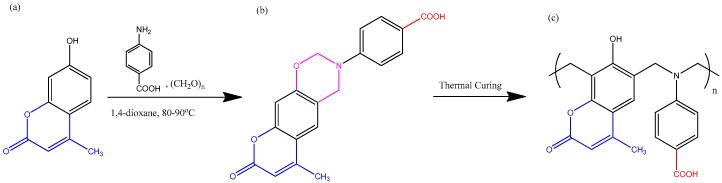
Chemical structures of: (**a**) Coumarin-OH; (**b**) Coumarin-COOH BZ; and (**c**) poly(Coumarin-COOH BZ).

In this study, we synthesized a new BZ monomer (Coumarin-COOH BZ), containing both coumarin and COOH units, through a facile Mannich condensation of 4-methyl-7-hydroxycoumarin (Coumarin-OH), 4-aminobenzoic acid, and paraformaldehyde in 1,4-dioxane (Scheme 1). We then blended this BZ monomer with homopolymeric PVP to form miscible poly(Coumarin-COOH BZ)/PVP blends after thermal curing. Because the photoresponsive coumarin units increased the crosslinking density and the COOH groups increased the number and strength of hydrogen bonding interactions with the C=O groups of PVP, the thermal properties of these blends were superior to those of traditional B-a–type polybenzoxazine/PVP blend systems [21]. We used differential scanning calorimetry (DSC), Fourier transform infrared (FTIR) spectroscopy, and thermogravimetric analysis (TGA) to monitor the thermal curing behavior and thermal stability of this new BZ monomer, both before and after photodimerization of the coumarin ring, and the hydrogen bonds between the Coumarin-COOH BZ and PVP moieties.

## 2. Experimental

### 2.1. Materials

4-Aminobenzoic acid (99%), paraformaldehyde (97%), and PVP (*M*_w_ = 58,000) were purchased from Alfa Aesar (Ward Hill, MA, USA). *N*,*N*-Dimethylformamide (DMF) and 1,4-dioxane were purchased from Acros Organic (St. Louis, MO, USA) and used without further purification. Coumarin-OH was synthesized according to a previously reported procedure [12].

#### 2.1.1. 4-(6-Methyl-8-oxochromeno[6,7-e][1,3]oxazin-3(2H,4H,8H)-yl)benzoic Acid (Coumarin-COOH BZ)

In a 250-mL two-neck round-bottom flask equipped with a stirring bar, Coumarin-OH (2.000 g, 11.36 mmol), 4-aminobenzoic acid (3.268 g, 23.86 mmol), and paraformaldehyde (0.7500 g, 24.99 mmol) were placed under vacuum for 10 min and then under a N_2_ atmosphere. 1,4-Dioxane (120 mL) was added and then the solution was heated to reflux at 80–90 °C with stirring. After 24 h, the solution was cooled to room temperature and the solids filtered. Then, the crude solid products were dissolved in dichloromethane (500 mL) under ultrasonic for 30 min and stirred for 1 h. Then, the solids filtered to give a slightly gray tinted solid (5.11 g, 85%). FTIR (KBr, cm^−1^): 1055 (C–O stretching), 1269 (asymmetric COC stretching), 1383 (CH_2_ wagging), 1680 (C=O stretching of COOH), 1734 (C=O stretching of coumarin moiety), 922 and 1518 (vibrations of trisubstituted benzene ring), 3053–2500 (COOH). ^1^H NMR (500 MHz, DMSO-*d*_6_, δ, ppm): 2.37 (s, 3H, CH_3_), 4.84 (s, 2H, CCH_2_N), 5.63 (s, 2H, OCH_2_N), 6.24 (s, 1H, CCH=C), 6.70–7.84 (m, 4H, CH aromatic), 12.38 (s, 1H, COOH). ^13^C NMR (125 MHz, DMSO-*d*_6_, δ, ppm): 18.16 (CH_3_), 44.18 (CCH_2_N), 77.97 (OCH_2_N), 111.15 (CCH=C), 112.96–156.90 (aromatic), 159.70 (C=O), 167.00 (COOH). High-resolution FT-MS: calcd. for [M + H]^+^ (C_19_H_16_NO_5_): *m*/*z* 338.33; found 338.10.

#### 2.1.2. Coumarin-COOH BZ/PVP Blends

In a 20-mL sample vial equipped with a stirring bar, desired weight amounts (weight ratios) of Coumarin-COOH BZ and PVP were dissolved in DMF (10 mL) and then mixed through ultrasonication (Delta-D150H, 43 kHz) for 4 h. After 72 h of stirred, the DMF was evaporated under vacuum to give a solid.

#### 2.1.3. Photodimerization of Coumarin-COOH BZ

A solution of Coumarin-COOH BZ (0.2 g) in CHCl_3_ (10 mL) was placed in a quartz tube and irradiated for 90 min at room temperature in a merry-go-round–type photoreactor equipped with five Philips lamps emitting light nominally at 365 nm.

#### 2.1.4. Poly(Coumarin-COOH BZ)/PVP Blends through Thermal Curing

Desired amounts of Coumarin-COOH BZ/PVP blends were placed onto aluminum pans and polymerized in a stepwise manner: at 150, 180, 210, and 240 °C for 2 h each. Each cured sample had a red color, which darkened as the curing temperature increased.

### 2.2. Characterization

^1^H and ^13^C nuclear magnetic resonance (NMR) spectra were recorded using a Varian UNITY INOVA 500 instrument (Palo Alto, CA, USA), with DMSO-*d*_6_ and CDCl_3_ as solvents and tetramethylsilane as the external standard. Chemical shifts are reported in parts per million (ppm). FTIR spectra of the polymer films were recorded using a Bruker Tensor 27 FTIR spectrophotometer (El Monte, CA, USA) and the conventional KBr disk method; 32 scans were collected at a spectral resolution of 4 cm^−1^; the prepared films were sufficiently thin to obey the Beer–Lambert law. Dynamic curing kinetics were determined using a TA Q-20 differential scanning calorimeter (Waters, Taipei, Taiwan) operated under a N_2_ atmosphere; the sample (ca. 5 mg) was placed in a sealed aluminum sample pan and dynamic curing scans were recorded from 30 to 350 °C at a heating rate of 20 °C min^−1^. The thermal stabilities of the samples were measured using a TA Q-50 thermogravimetric analyzer (Waters, Taipei, Taiwan) operated under a N_2_ atmosphere; a cured sample (ca. 5 mg) was placed in a Pt cell and heated at a rate of 20 °C min^−1^ from 30 to 800 °C under a N_2_ flow of 60 mL min^−1^.

## 3. Results and Discussion

### 3.1. Synthesis of Coumarin-COOH BZ through Mannich Reaction of Coumarin-OH with 4-Aminobenzoic Acid and Paraformaldehyde

**Figure 1:**
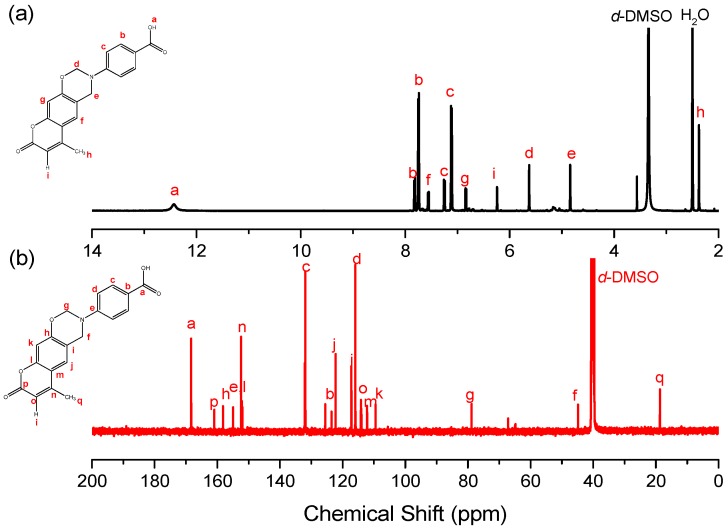
(**a**) ^1^H; and (**b**) ^13^C NMR spectra of Coumarin-COOH BZ.

**Figure 2:**
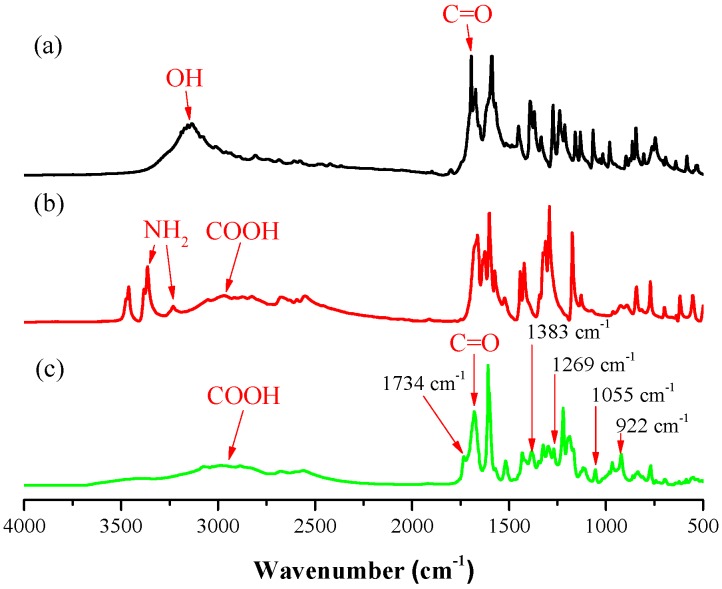
FTIR spectra of: (**a**) Coumarin-OH; (**b**) 4-aminobenzoic acid; and (**c**) Coumarin-COOH BZ.

We prepared a new BZ monomer containing both a COOH group and a coumarin moiety, with the expectation that the latter, upon UV (365 nm) exposure, would undergo photodimerization through [2π + 2π] cycloaddition to enhance the thermal stability (glass transition temperature, degradation temperature, and char yield) of resulting PBZ polymers. In addition, we expected the COOH moiety of the monomer to serve an important role by facilitating hydrogen bonding within this system. First, we synthesized Coumarin-OH according to procedures we have reported previously [12]. Next, we obtained Coumarin-COOH BZ from the Mannich condensation of Coumarin-OH, 4-aminobenzoic acid, and paraformaldehyde in 1,4-dioxane at 80–90 °C (Scheme 1). ^1^H NMR spectrum of Coumarin-COOH BZ monomer (Figure 1a) provided two peaks at 4.84 and 5.63 ppm corresponding to its CCH_2_N and OCH_2_N units, respectively. The ^13^C NMR spectrum of Coumarin-COOH BZ monomer (Figure 1b) displays the characteristic peaks appear at 44.18 and 77.97 ppm, representing the C**CH**_2_N and O**CH**_2_N units, respectively, of its oxazine ring structure. Figure 2 presents the FTIR spectra of Coumarin-OH, 4-aminobenzoic acid, and Coumarin-COOH BZ at room temperature. The spectrum of Coumarin-OH exhibits a sharp band for the C=O group at 1695 cm^−1^ and a broad band for the OH group at 3200 cm^−1^ (Figure 2a). The spectrum of 4-aminobenzoic acid (Figure 2b) features two sharp signals for the amino group at 3200–3460 cm^−1^ and a broad band for the COOH group at 2551–3051 cm^−1^. The spectrum of Coumarin-COOH BZ (Figure 2c) exhibits several characteristic bands at 1055 (C–O stretching), 1269 (asymmetric COC stretching), 1680 and 1734 (C=O stretching), 2500–3053 (COOH), and 922 and 1518 (vibration of a trisubstituted benzene ring) cm^−1^. These ^1^H and ^13^C NMR and FTIR spectra are consistent with the successful synthesis of Coumarin-COOH BZ.

### 3.2. Thermal Polymerization of Coumarin-COOH BZ

**Figure 3:**
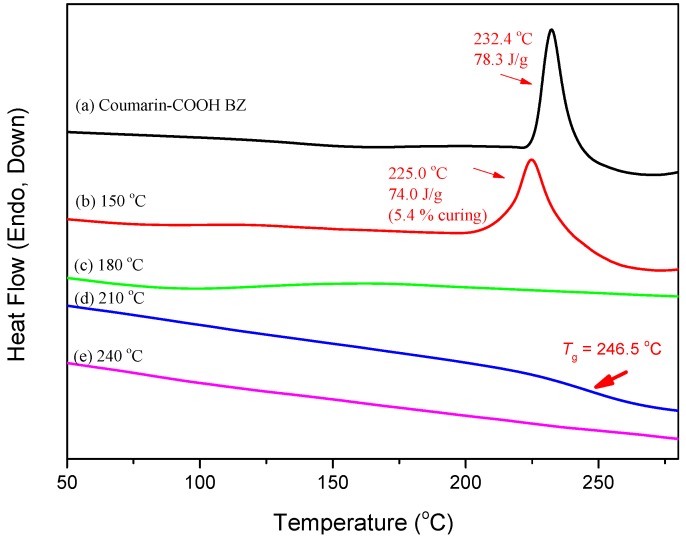
DSC thermograms of Coumarin-COOH BZ: (**a**) uncured monomer; and (**b**–**e**) after thermal curing at: (**b**) 150; (**c**) 180; (**d**) 210; and (**e**) 240 °C, recorded after each curing stage.

We employed FTIR spectroscopy, DSC, and TGA to investigate the thermal polymerization of the Coumarin-COOH BZ monomer. Figure 3 presents DSC thermograms, recorded at a heating rate of 20 °C min^−1^, of Coumarin-COOH BZ that had been subjected to various curing temperatures. The DSC trace of the uncured Coumarin-COOH BZ monomer exhibited a sharp exothermic curve with a maximum at 232.4 °C, and a reaction heat of 78.3 J g^−1^. The enthalpies of the exotherms of Coumarin-COOH BZ decreased upon increasing the curing temperature. The exothermic peak vanished completely when the curing temperature was above 180 °C, indicating that the polymerization of Coumarin-COOH BZ was complete at a temperature higher than 180 °C. In addition, the glass transition temperature of Coumarin-COOH BZ was found at 246.5 °C when the thermal curing temperature was at 210 °C. When the curing temperature increased to 240 °C, the glass transition temperature was not found, probably due to the high crosslinking density or the glass transition temperature being too high to measure in this case. Hence, the curing temperature setting at 210 °C for Coumarin-COOH BZ was reasonable for the further experiments in this study. Furthermore, the risk of material decomposition would be considered as the curing temperature increases.

**Figure 4:**
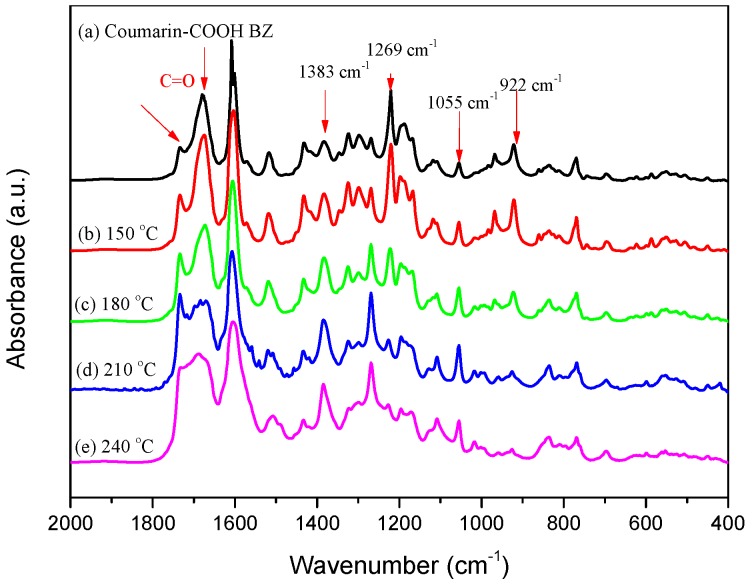
FTIR spectra of Coumarin-COOH BZ: (**a**) uncured monomer; and (**b**–**e**) after thermal curing at: (**b**) 150; (**c**) 180; (**d**) 210; and (**e**) 240 °C, recorded after each curing stage.

Figure 4 presents corresponding FTIR spectra, recorded at ambient temperature, of Coumarin-COOH BZ samples that had experienced various curing temperatures. The intensities of the characteristic bands of Coumarin-COOH BZ at 1269 cm^−1^ (asymmetric COC stretching of oxazine) and 922 cm^−1^ (C–H out-of-plane bending) all decreased gradually upon increasing the curing temperature, which is consistent with the DSC analyses.

**Figure 5:**
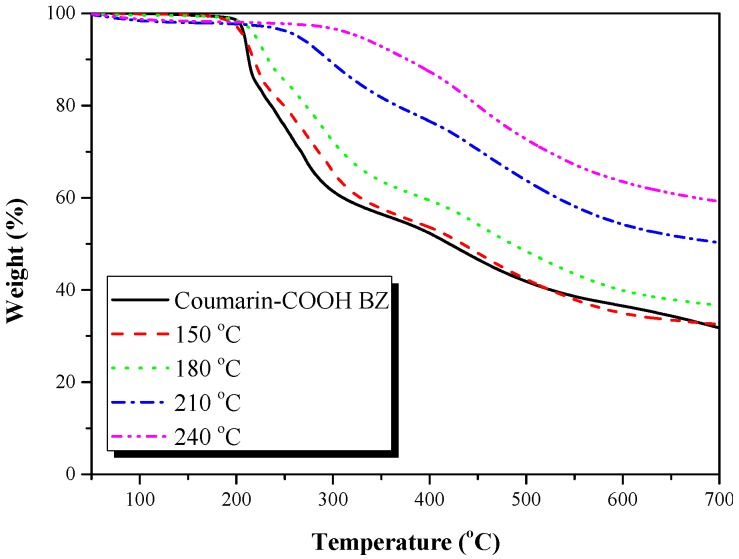
TGA analyses of Coumarin-COOH BZ, recorded after each curing stage.

Figure 5 presents the thermal stabilities (TGA under a N_2_ atmosphere) of Coumarin-COOH BZ sample that had been subjected to various curing temperatures. Clearly, three steps of decomposition reaction occur at various temperatures during TGA measurements. Firstly, the Mannich base cleavage results in amine evaporation while the temperature is below 300 °C. The second and third main weight losses are possibly attributable to the decomposition of phenol (300 to 400 °C) and coumarin moieties (at 400 °C), respectively [28]. We measured the temperature at which the weight loss reached 10% (*T*_d_) as our standard. Both the value of *T*_d_ and the char yield increased upon increasing the curing temperature (e.g., uncured sample: *T*_d_ = 213 °C, Char yield = 31 wt %; thermal curing sample after 240 °C treatment: *T*_d_ = 374 °C, char yield = 60 wt %), implying that the crosslinking density was increased as the curing temperature increased, thereby enhancing the thermal stability of poly(Coumarin-COOH BZ).

### 3.3. Thermal Polymerization of Coumarin-COOH BZ/PVP Blends

**Figure 6:**
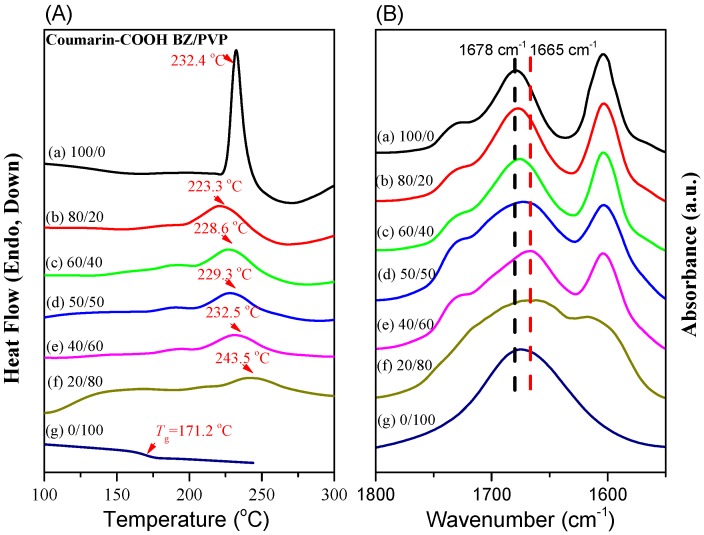
(**A**) DSC thermograms; and (**B**) FTIR spectra of Coumarin-COOH BZ/PVP blends before thermal curing: (**a**) 100/0; (**b**) 80/20; (**c**) 60/40; (**d**) 50/50; (**e**) 40/60; (**f**) 20/80; and (**g**) 0/100.

We employed DSC and FTIR spectroscopy to investigate the properties of Coumarin-COOH BZ blends with various amounts of PVP. Figure 6A presents the DSC trace of the uncured Coumarin-COOH BZ/PVP blend system; we find that the thermal curing temperature was affected after adding the PVP homopolymer. At a low PVP concentration (20 wt %), the temperature of the exothermic peak decreased comparing with the pure Coumarin-COOH BZ. The temperature of the exothermic peaks, however, increased upon increasing the PVP concentration because of inhibited ring opening thermal polymerization of the BZ rings. Figure 6B displays the FTIR spectra (1800–1550 cm^−1^ region), recorded at room temperature, of the various Coumarin-COOH BZ/PVP blends. Three major peaks at 1732, 1678, and 1603 cm^−1^ appeared for pure Coumarin-COOH BZ, representing the C=O group of the coumarin unit, the carboxylic acid (COOH) group, and the aromatic ring, respectively. The broad band of pure PVP at 1678 cm^−1^ represented its free C=O stretching bands (Figure 6B-g). Because the signals of the carboxylic acid of Coumarin-COOH BZ and the free C=O groups of PVP overlapped considerably, it was difficult to calculate the exact fraction of the C=O groups of PVP that were hydrogen bonded with Coumarin-COOH BZ; however, we could clearly observe that the signal for the hydrogen-bonded C=O groups of PVP shifted to lower wavenumber (1665 cm^−1^) for the Coumarin-COOH BZ/PVP = 20/80, 40/60, and 50/50 blend system. Thus, hydrogen bonding interactions within the Coumarin-COOH BZ/blend assisted in preventing phase separation after thermal curing of the BZ ring.

**Figure 7:**
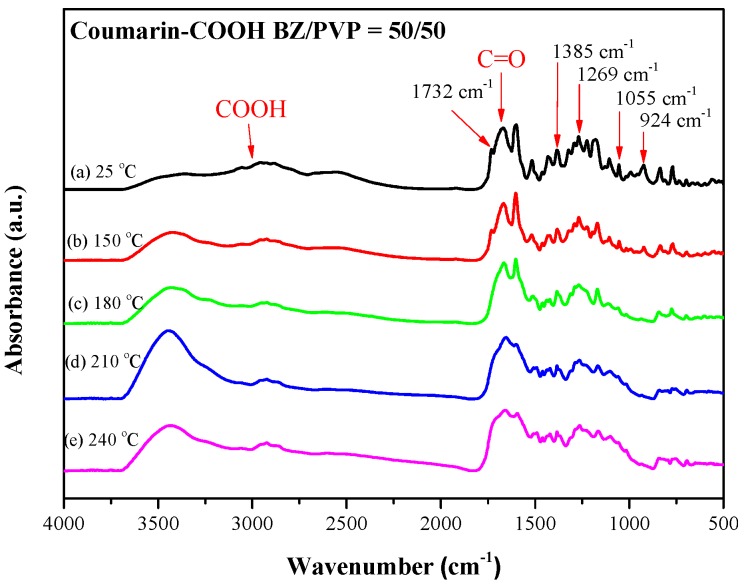
FTIR spectra of the Coumarin-COOH BZ/PVP = 50/50 blend: (**a**) uncured monomer; and (**b**–**e**) after thermal curing at: (**b**) 150; (**c**) 180; (**d**) 210; and (**e**) 240 °C, recorded after each curing stage.

Figure 7 presents the corresponding FTIR spectra of the Coumarin-COOH BZ/PVP = 50/50 blend after curing at various temperatures. The intensity of the signal for C–H out-of-plane bending (924 cm^−1^) decreased gradually upon increasing the curing temperature; this characteristic band disappeared completely after curing at 210 °C. Thus, for our subsequent studies, we subjected all Coumarin-COOH BZ/PVP blends to thermal curing at 210 °C to form poly(Coumarin-COOH BZ)/PVP blend systems.

**Figure 8:**
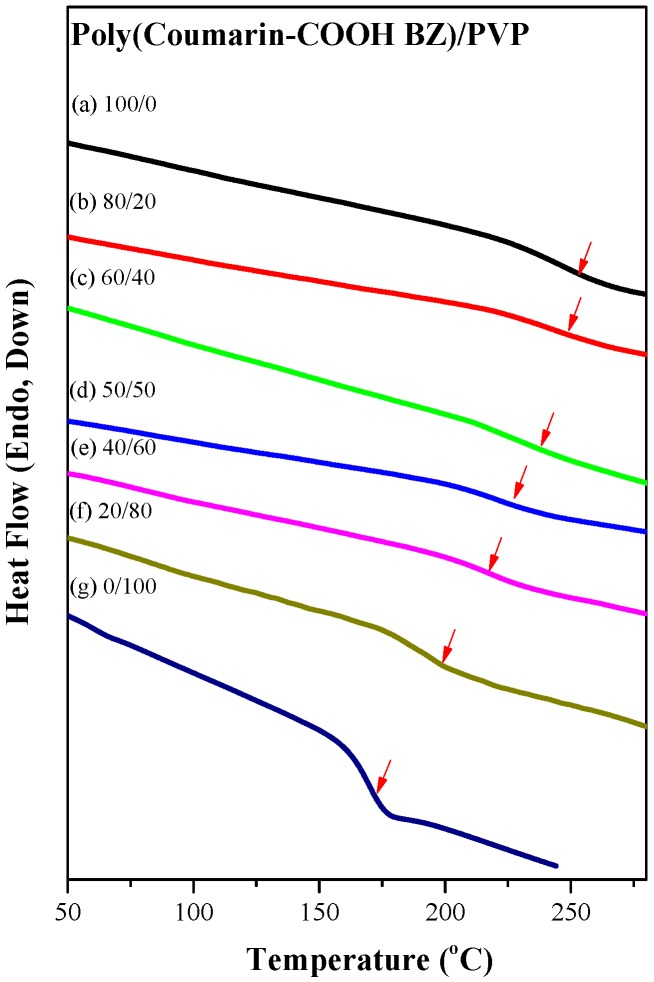
DSC thermograms of poly(Coumarin-COOH BZ)/PVP blends: (**a**) 100/0; (**b**) 80/20; (**c**) 60/40; (**d**) 50/50; (**e**) 40/60; (**f**) 20/80; and (**g**) 0/100.

Based on DSC analyses, Figure 8 displays the dependence of the value of *T*_g_ on the composition of the poly(Coumarin-COOH BZ)/PVP blends. Pure poly(Coumarin-COOH BZ) and pure PVP provided values of *T*_g_ of 246.5 and 171.4 °C, respectively. A single value of *T*_g_ appeared for all poly(Coumarin-COOH BZ)/PVP blend systems, indicating that they were all miscible in the amorphous phase.

**Figure 9:**
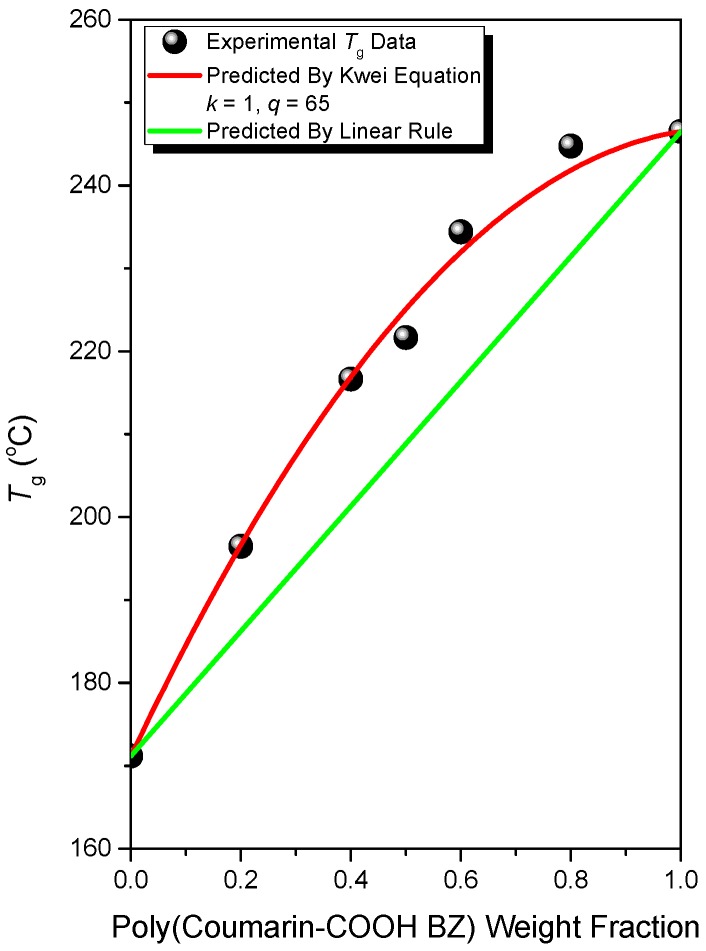
Glass transition temperature/composition curves, based on the Kwei equation, for poly(Coumarin-COOH BZ)/PVP blends.

More interestingly, this single value of *T*_g_ exhibited a large positive deviation from the linear rule (Figure 9), implying that strong hydrogen bonding was present in this blend system. In general, the Kwei equation suggests the following relationship for the value of *T*_g_ for a miscible polymer blend featuring strong hydrogen bonding interactions [30]:

(1) $$T_{g}=\frac{W_{1}T_{g1}+kW_{2}T_{g2}}{W_{1}+kW_{2}}+qW_{1}W_{2}$$

where *W*_1_ and *W*_2_ represent the weight fractions of poly(Coumarin-COOH BZ) and PVP, respectively; *T*_g1_ and *T*_g2_ are the glass transition temperatures of pure poly(Coumarin-COOH BZ) and pure PVP, respectively; and *k* and *q* are fitting constants. After fitting our data to the Kwei equation (solid line), we obtained values for *k* and *q* of 1 and 65, respectively (Figure 8), consistent with strong intermolecular hydrogen bonding between poly(Coumarin-COOH BZ) and PVP. Although thermal curing of the BZ ring would significantly increase the molecular weight and, thereby, decrease the entropy of mixing in Gibbs free energy, strong hydrogen bonding existed to prevent phase separation in the thermoset/thermoplastic blend system. Compared with PBZ/PVP blend system, the poly(Coumarin-COOH BZ)/PVP shows higher *T*_g_ behavior at all blend compositions. For example, the poly(Coumarin-COOH BZ)/PVP = 50/50 blend exhibits *T*_g_ value at 221 °C and PBZ/PVP shows the *T*_g_ value at 188 °C, which is the advantage of functionalization in this work.

**Figure 10:**
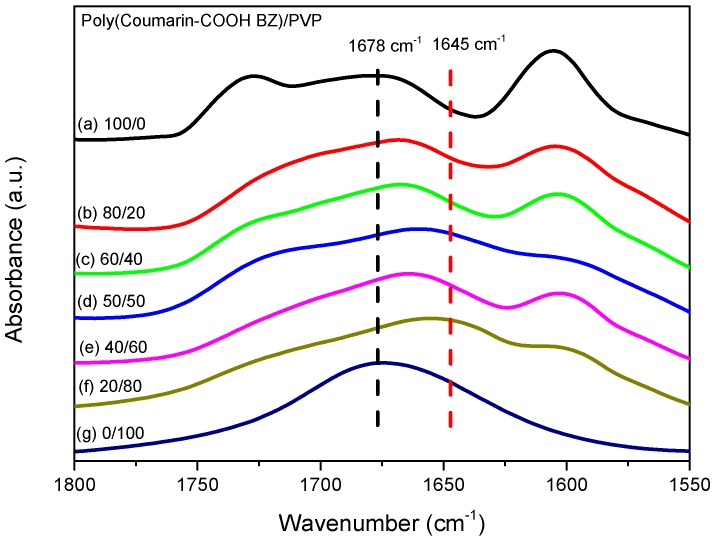
FTIR spectra (1550–1800 cm^−1^), recorded at room temperature, for poly(Coumarin-COOH) BZ/PVP blends: (**a**) 100/0; (**b**) 80/20; (**c**) 60/40; (**d**) 50/50; (**e**) 40/60; (**f**) 20/80; and (**g**) 0/100.

Figure 10 displays FTIR spectra, recorded at room temperature, of various poly(Coumarin-COOH BZ)/PVP blends after thermal curing. Thermal curing of the Coumarin-COOH BZ monomer resulted in an extra phenolic OH group appearing (as shown in Scheme 1c) that would also interact with the C=O groups of PVP to form miscible poly(Coumarin-COOH BZ)/PVP blends. Indeed, a significant wavenumber shift, from 1678 to 1645 cm^−1^, occurred after thermal curing of the Coumarin-COOH BZ monomer, consistent with stronger hydrogen bonding occurring after thermal curing.

### 3.4. Photodimerization of Coumarin Moieties Through [2π + 2π] Cycloaddition

**Scheme 2:**
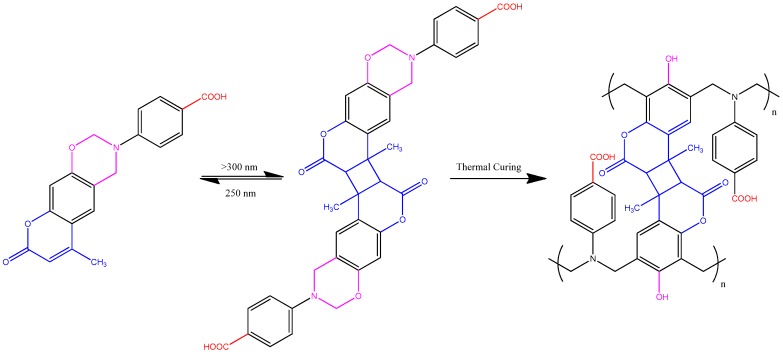
Photodimerization of Coumarin-COOH BZ with subsequent thermal curing to form PBZ matrices of high crosslinking density.

Under UV irradiation, coumarin moieties can undergo photochemical dimerization, as shown in Scheme 2.

**Figure 11:**
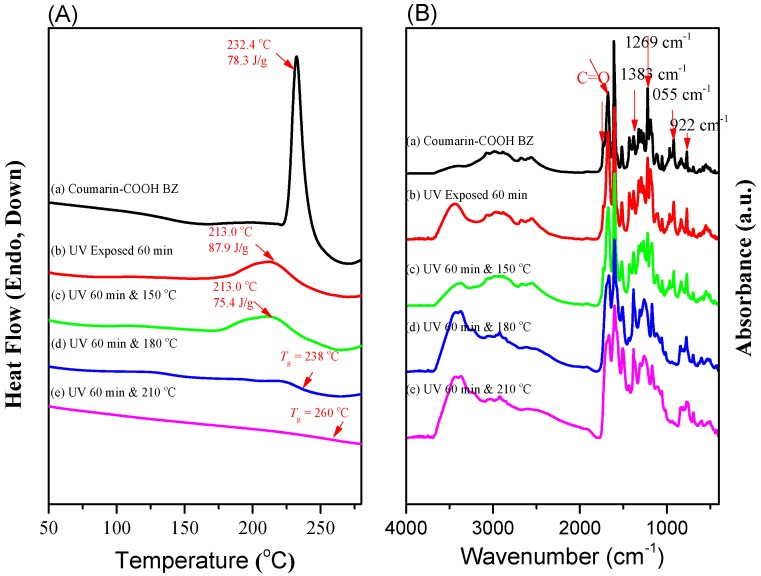
(**A**) DSC thermograms; and (**B**) FTIR spectra of Coumarin-COOH BZ: (**a**) uncured monomer; (**b**) after irradiation at 365 nm; and (**c**–**e**) after irradiation at 365 nm and thermal curing at: (**c**) 150; (**d**) 180; and (**e**) 210 °C, recorded after each curing stage.

Figure 11A presents DSC thermograms of Coumarin-COOH BZ before and after UV exposure, recorded upon a heating stage at a heating rate of 20 °C min^−1^. After UV exposure at 365 nm for 60 min, the temperature of the exothermic peak shifted from 232.4 to 213.0 °C, presumably because of destruction of the surface during irradiation (Figure 11A-b). The enthalpies of the exotherms of Coumarin-COOH BZ after UV exposure decreased upon increasing the curing temperature. The exothermic peak vanished completely until the curing temperature was above 180 °C, indicating that the polymerization of Coumarin-COOH BZ was completed at 180 °C. Figure 11B displays the corresponding FTIR spectra of Coumarin-COOH BZ before and after UV exposure. The signal at 922 cm^−1^, corresponding to C–H out-of-plane bending, decreased gradually upon increasing the curing temperature; its characteristic bands disappeared completely at 180 °C, consistent with the DSC analysis. In addition, the glass transition temperature of Coumarin-COOH BZ was 260.0 °C when the curing temperature was 210 °C, higher than that (246.5 °C) of its uncured counterpart, consistent with an increased crosslinking density for the PBZ after photodimerization of the coumarin units.

**Figure 12:**
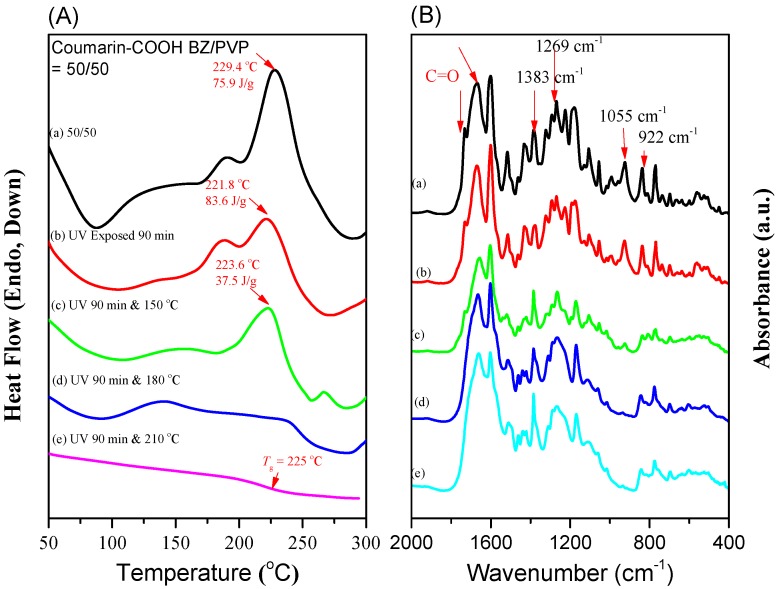
(**A**) DSC thermograms; and (**B**) FTIR spectra of the Coumarin-COOH BZ/PVP = 50/50 blend: (**a**) uncured monomer; (**b**) after irradiation at 365 nm; and (**c**–**e**) after irradiation at 365 nm and thermal curing at: (**c**) 150; (**d**) 180; and (**e**) 210 °C, recorded after each curing stage.

Figure 12A presents DSC thermograms of the Coumarin-COOH BZ/PVP = 50/50 blend before and after UV exposure, recorded on a heating stage at a heating rate of 20 °C min^−1^. The curing behavior became complicated after blending with the PVP homopolymer, with the two major peaks observed for Coumarin-COOH BZ/PVP = 50/50 at 229.4 and 190.0 °C. The curing temperatures for the blend system shifted from 232.4 °C for pure Coumarin-COOH BZ, presumably because of the basicity of the pyrrolidine (N–C=O) group, suggesting that this functional group could act as a basic catalyst to lower the thermal curing temperature. Similar to the situation for the Coumarin-COOH BZ/PVP = 50/50 blend system, the temperature of the exothermic peak shifted from 229.4 to 221.8 °C after UV exposure at 365 nm for 90 min, possibly because of destruction of the surface during irradiation (Figure 12A). The enthalpies of the exotherms of the Coumarin-COOH BZ/PVP = 50/50 blend after UV exposure decreased upon increasing the curing temperature. The exothermic peak vanished completely when the curing temperature reached 180 °C, suggesting that the polymerization of the blend composites of Coumarin-COOH BZ/PVP = 50/50 was complete at a temperature lower than 180 °C; in addition, the signal at 922 cm^−1^, representing C–H out-of-plane bending, disappeared completely after curing at 180 °C (Figure 12B), consistent with the DSC analysis. Furthermore, the value of *T*_g_ of the Coumarin-COOH BZ/PVP = 50/50 blend system after UV exposure (225.0 °C) was higher than that (221.6 °C) of the thermally cured sample that had not been subjected to UV exposure, again due to the increased crosslinking density of the PBZ after photodimerization of the coumarin units.

**Figure 13:**
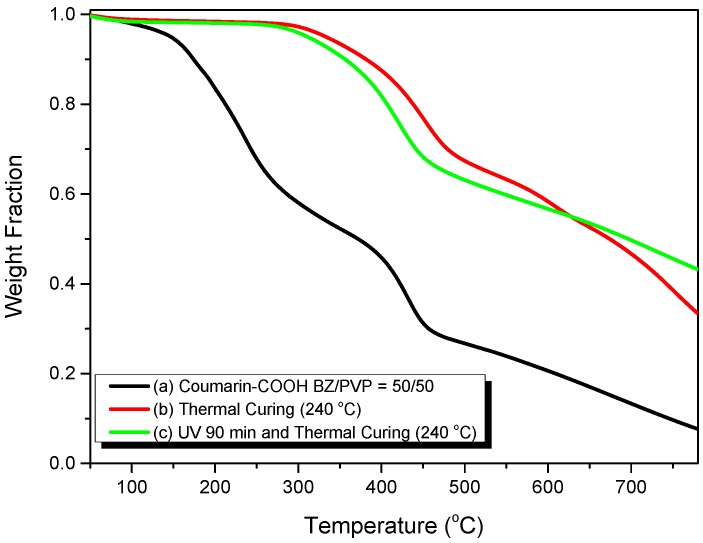
TGA analyses of the Coumarin-COOH BZ/PVP = 50/50 blend: (**a**) uncured monomer; (**b**) after thermal curing; and (**c**) after irradiation at 365 nm and thermal curing.

Figure 13 presents TGA thermograms of the uncured and thermally cured (at 240 °C) Coumarin-COOH BZ/PVP = 50/50 blends before and after UV exposure under a N_2_ atmosphere. Similar three steps of decompose reactions was found, which is similar with Figure 5. The values of *T*_d_ for the uncured Coumarin-COOH BZ/PVP = 50/50 blend and for the blend thermally cured at 240 °C, before and after photodimerization, were 174.1, 381.3, and 356.2 °C, respectively; the char yields were 6.9%, 31.6%, and 42.3%, respectively. Thus, the values of char yields increased after both thermal curing and UV exposure, suggesting that the crosslinking density had increased upon increasing the curing temperature and UV exposure to enhance the thermal stability of the Coumarin-COOH BZ/PVP blend system. However, the value of *T*_d_ decreased after UV exposure, presumably because of destruction on the surface during irradiation.

## 4. Conclusions

We have prepared a novel bifunctional BZ monomer possessing both coumarin and COOH moieties. DSC revealed a single value of *T*_g_ for each Coumarin-COOH BZ/PVP blend, but with a large positive deviation, based on the Kwei equation, because of strong hydrogen bonding between the Coumarin-COOH BZ and PVP segments. The thermal properties (e.g., glass transition and thermal degradation temperatures) of the blends improved as a result of the increased crosslinking density after photodimerization under UV exposure.
